# Progressive Hydrogel Applications in Diabetic Foot Ulcer Management: Phase-Dependent Healing Strategies

**DOI:** 10.3390/polym17172303

**Published:** 2025-08-26

**Authors:** Priyanka Mallanagoudra, Sai Samanvitha M Ramakrishna, Sowmya Jaiswal, Dhruthi Keshava Prasanna, Rithika Seetharaman, Arunkumar Palaniappan, Sudarshan Kini

**Affiliations:** 1 Nitte (Deemed to be University), Department of Bio & Nano Technology, Nitte University Centre for Science Education and Research, Mangalore 575018, Karnataka, India; priyanka.23pbos103@student.nitte.edu.in (P.M.); samanvithamr@gmail.com (S.S.M.R.); sowmyajaiswal28@gmail.com (S.J.); dhruthikp03@gmail.com (D.K.P.); rithikame1006@gmail.com (R.S.); 2 Human Organ Mimics Engineering (HOME) Labs, Centre for Biomaterials, Cellular and Molecular Theranostics (CBCMT), Vellore Institute of Technology, Vellore 632014, Tamil Nadu, India; arunkumar@dubai.bits-pilani.ac.in; 3 Department of Biotechnology, Birla Institute of Technology and Science, Pilani, Dubai Campus, Dubai 345055, United Arab Emirates

**Keywords:** diabetic foot ulcer, wound healing, hydrogel, microenvironment, inflammation

## Abstract

Diabetes is emerging as a significant health and societal concern globally, impacting both young and old populations. In individuals with diabetic foot ulcers (DFUs), the wound healing process is hindered due to abnormal glucose metabolism and chronic inflammation. Minor injuries, blisters, or pressure sores can develop into chronic ulcers, which, if left untreated, may lead to serious infections, tissue necrosis, and eventual amputation. Current management techniques include debridement, wound dressing, oxygen therapy, antibiotic therapy, topical application of antibiotics, and surgical skin grafting, which are used to manage diabetic wounds and foot ulcers. This review focuses on a hydrogel-based strategy for phase-wise targeting of DFUs, addressing sequential stages of diabetic wound healing: hemostasis, infection, inflammation, and proliferative/remodeling phases. Hydrogels have emerged as a promising wound care solution due to their unique properties in providing a suitable wound-healing microenvironment. We explore natural polymers, including hyaluronic acid, chitosan, cellulose derivatives, and synthetic polymers such as poly (ethylene glycol), poly (acrylic acid), poly (2-hydroxyethyl methacrylate, and poly (acrylamide), emphasizing their role in hydrogel fabrication to manage DFU through phase-dependent strategies. Recent innovations, including self-healing hydrogels, stimuli-responsive hydrogels, nanocomposite hydrogels, bioactive hydrogels, and 3D-printed hydrogels, demonstrate enhanced therapeutic potential, improving patient outcomes. This review further discusses the applicability of various hydrogels to each phase of wound healing in DFU treatment, highlighting their potential to advance diabetic wound care through targeted, phase-specific interventions.

## 1. Introduction

Diabetes mellitus, a pervasive and intricate chronic ailment, is a global health predicament with immense prevalence. An estimated 537 million adults globally, or 10.5% of all adults ranging between 29 and 70 years, have diabetes. Globally, the number of people with diabetes is projected to increase from 643 million in 2030 to 783 million in 2045 [1]. This metabolic disorder disrupts the intricate balance of glucose regulation in the body, leading to increased blood sugar levels, which can have extensive ramifications for an individual’s well-being and health. Given that diabetes has two primary types and that millions of people worldwide suffer from both Type-1 and Type-2 diabetes, the disease’s effects extend beyond one’s health to include societal, economic, and healthcare-related ramifications. It is essential to understand the primary causes and treatments of diabetes mellitus to properly manage this prevalent condition and improve the quality of life of affected individuals. Diabetes, a chronic endocrine and metabolic disease, is a significant initiator of many other co-occurring health consequences, such as cardiovascular diseases, mental illnesses, kidney diseases, eye disorders, neuropathy, rheumatoid arthritis, and osteopathic diseases [2].

Diabetic foot ulcer (DFU) causes complications due to slow-healing wounds in individuals with diabetes. Ulcers are open wounds that often develop on the feet, especially on the soles or sides. Approximately 5% of patients with diabetes mellitus face the emergence of foot ulcers, and 1% of these progress to a severe outcome of amputation [3]. Diabetes can lead to peripheral vascular disease and neuropathy, which reduces blood flow to the limbs, increases the risk of injury, and delays recovery in the foot. This can result in serious infections, tissue damage, and inflammation of the bone, also called osteitis, eventually leading to gangrene and posing the risk of amputation. Supplementary Figure S1 shows the development of foot ulcers in diabetics. Initially, superficial injuries such as small cuts or scratches acquire greater significance in diabetic patients, where these injuries tend to progress rapidly into ulcers due to peripheral neuropathy and impaired vascular supply [4]. In advanced stages, partial or total gangrene may occur due to blood flow obstruction and necrotic tissue formation. The infection may also spread to the bone, complicating the clinical picture further. This progression necessitates vigilant foot care, regular monitoring, and timely therapeutic interventions for diabetic patients to prevent severe consequences like limb loss [3].

Preventing, identifying, and treating DFUs is essential to minimizing their harmful effects. The leading cause of DFUs is high blood sugar levels, which damage the blood vessels in the feet and increase inflammation. High blood sugar levels also constrict blood vessels and alter the membrane of red blood cells, reducing the oxygen supply, which leads to diabetic neuropathy. The detrimental effects of hyperglycemia on wound healing are apparent in the proliferative phase. Bacterial infections and biofilm formation further complicate the wound environment. These factors hinder the normal healing process and result in nonhealing chronic wounds [5].

The choice of wound dressings for DFUs generally relies on the extent of the ulcer and the phases of the healing mechanism. Examples of commercially available wound-dressing hydrogel patches are listed in Table 1. However, prevailing wound dressings frequently encounter significant complications such as deficient moisture regulation, insufficient wound bed preparation, restricted antimicrobial attributes, and inadequate stimulation potential for healing. Thus, there is a strong need for better and more sophisticated wound dressings to effectively address the pressing concerns associated with DFU within the patient community.

Hydrogels represent an exceptional category of cross-linked polymeric networks characterized by their three-dimensional structures. These networks possess the distinctive ability to retain significant quantities of both aqueous solvents and biological fluids within their frameworks [6]. Currently employed hydrophilic polymers, such as hyaluronic acid (HA), poly (ethylene glycol) (PEG), and peptides, as well as zwitterionic polymers such as polycarboxybetaine (PCB), polysulfobetaine (PSB), poly (quaternized triazole carboxybetaine acrylamide) [P(qTR-CB)], and poly (2-methacryloyloxyethyl phosphorylcholine) (PMPC), are recognized for their excellent antifouling properties, water-binding capability, and exceptional superhydrophilicity [7]. Mabrouk et al. fabricated a three-layered wound patch using poly (acrylic acid) (PAA), polyvinyl pyrrolidone (PVP), and polycaprolactone (PCL) to treat DFU. The polymer PAA helps maintain moisture in the wound area, whereas PVP allows enhanced drug holding and release. PCL provides mechanical strength to the hydrogel and helps in slow degradation, which helps extend the treatment period. The resulting patches exhibited improved mechanical properties and favorable wettability and adherence, thus demonstrating promising potential for effective DFU treatment [8]. HA-based hydrogels such as Restore^®^ and Regenecare^®^ are favored for their low antigenicity and superior moisturizing properties that promote skin repair [9].

Chronic wounds are persistent, especially when associated with underlying conditions, such as diabetes and other vascular diseases. Therefore, novel treatment approaches are required. The use of hydrogels in the management of DFU presents an avenue with considerable potential for addressing these challenging wounds. Several characteristics of hydrogels, whether natural or synthetic, contribute to their biodegradability, antimicrobial and anti-inflammatory properties, ability to sustain a moist environment, deliver bioactive molecules, and promote tissue regeneration and angiogenesis [10]. In this review, we begin by contrasting the healing mechanisms of a typical wound with those of a DFU wound to highlight the specific challenges posed by the DFU microenvironment. Understanding the complexities of the wound microenvironment is essential for effectively applying hydrogels at various stages of the wound healing process. Further, it explores the progress in hydrogel technology for DFU treatment, describing the unique attributes that enhance the effectiveness of hydrogels.

## 2. Normal Versus Dysregulated Wound Healing

**Figure 1:**
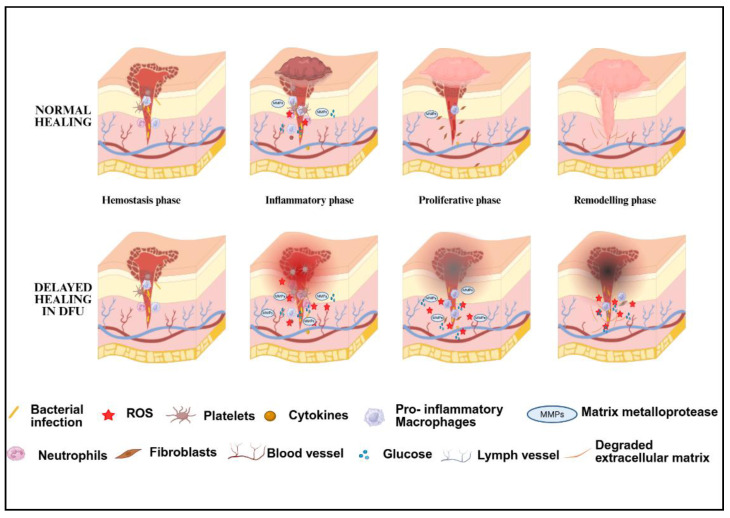
**Normal versus DFU healing:** Normal Wound healing involves hemostasis, inflammation, proliferation, and remodeling. In DFU, delayed clotting, prolonged inflammation, impaired proliferation, and poor collagen formation contribute to delayed wound healing. (Image source: Created with BioRender.com).

Normal wound healing typically proceeds through coordinated sequential events, each indispensable for restoring tissue integrity. Distinct from acute wounds, DFU is characterized by persistent inflammation that lasts for a prolonged period, typically exceeding four weeks in a clinical setting. While the healing process of DFU wounds shares similarities with that of normal acute wounds, it tends to reach a point where progress becomes stagnant. The key events of the wound healing process are as follows. The hemostasis phase of wound healing involves vasoconstriction, platelet aggregation, and recruitment of coagulation factors [11]. When a wound is sustained, neutrophils from the injured blood vessels recruit passively, initiating the inflammatory response. Alongside, local immune cells become activated, increasing the levels of chemokines, ROS, and inflammatory cytokines. Local endothelial activation resulting from these signals promotes vasodilation, increasing vascular permeability. Nitric oxide (NO), linked with inflammation, triggers this process. Furthermore, neutrophils release neutrophil extracellular traps (NETs), while macrophages secrete metalloproteinases (MMPs). The next stage of wound repair involves the proliferation and migration of various cells to the injury site. Keratinocytes secrete epidermal growth factor (EGF), signaling their proliferation and migration, followed by the sprouting of blood vessels (angiogenesis) and macrophage activation. During this phase, inflammation begins to reside. Fibroblasts secrete fibroblast growth factors (FGF2), and vascular endothelial cells secrete vascular endothelial growth factor (VEGF) to remodel and deposit new tissue through the extracellular matrix and promote neovascularization [11,12]. Further, the granular tissue forms a substrate for the migration of mature keratinocytes for the repair process. Some of the fibroblasts differentiate into myofibroblasts, which, upon interaction, produce collagen, ultimately leading to scar formation. The final stage of remodeling of the wound tissue involves the apoptosis of macrophages, myofibroblasts, and endothelial cells formed during the proliferation stage, leading to scar regression. The MMPs released by these cells remodel the type III collagen to type I collagen, which helps strengthen the repaired tissue [11,13]. The difference between normal wound healing and DFU healing phases is illustrated in Figure 1. The following section describes the sequential events of dysregulated wound healing associated with diabetes.

### 2.1. Hemostasis

The initial response to damage involves vasoconstriction in blood vessels and platelet clot formation, leading to coagulation. Fibrin from fibrinogen forms a temporary extracellular matrix (ECM). Activated cells release platelet-derived growth factor (PDGF) and transforming growth factor β (TGF-β) [5,14]. The processes involved in wound closure are influenced by individuals diagnosed with diabetes mellitus (DM). This influence is initiated by a reduction in fibrinolysis and an imbalance of cytokines, ultimately leading to an alteration in the closure of wounds. Moreover, patients with DM exhibit notable changes in the hemostasis phase, including hypercoagulability and decreased fibrinolysis, which further contribute to the complexity of wound closure in these individuals [14,15].

### 2.2. Inflammatory Phase

In response to damage or infection, inflammation occurs with the release of cytokines (IL-1, TNF-α, IL-6, IFN-γ) and growth factors (PDGF, TGF-β, IGF-1, EGF) by neutrophils, mast cells, and macrophages [5,16]. An imbalance of cytokines in individuals with DM significantly impacts wound repair. Studies have provided evidence indicating that neutrophils, a crucial component of the inflammatory response, exhibit an abnormal pattern of cytokine release and a decrease in their functionality in the context of DM. Due to hyperglycemia, the neutrophil protein arginine deiminase (PAD)-4 expression is upregulated. Neutrophils undergo histone citrullination upon activation by PAD-4 during NETosis, which leads to exocytosis and death of the neutrophil-PAD complex by NETs. While this mechanism engulfs the bacteria to protect against infection, releasing NETosis can impede the healing of damaged tissue. Nonhealing diabetic wounds have higher nuclear components like histones H2A, H2B, and H3 and characteristic granule components of NETs like neutrophil protease 3, neutrophil elastase, and myeloperoxidase [11]. Another major factor that impairs the DFU inflammatory phase is macrophages. Typically, macrophages (M1 polarized) secrete proinflammatory cytokines in the initial inflammatory stage. However, switching from M1 to M2 polarization allows macrophages to promote anti-inflammatory activity [11]. In the case of DFU, these proinflammatory factors fail to produce anti-inflammatory activity due to macrophage dysfunction, which leads to chronic inflammation. Macrophage function is impaired due to a loss of phagocytic ability by altering the advanced glycation end products (AGEs). AGEs interfere with macrophage receptor function, impairing their ability to recognize and engulf pathogens and debris effectively [4,17]. Vasoconstriction due to vascular dysfunction and insufficient oxygen delivery to the wounded area impairs the leukocyte migration and increases the risk of infection [18]. This inflammation persists for longer, disrupting tissue regeneration [14,19].

### 2.3. Proliferative Phase

This phase involves tissue repair and regeneration by fibroblasts producing collagen and other structural proteins. However, hyperglycemia and increased inflammation, particularly increased AGEs, lead to impaired fibroblast function that results in decreased proliferation. Angiogenesis creates new blood vessels, supplying oxygen and nutrients. Usually, angiogenesis takes place in hypoxic conditions. The wounds in healthy individuals undergo angiogenesis by recruiting Hypoxic inducible factors (HIF-1). This HIF-1 has two subunits, alpha (α) and beta (β); the HIF-1α degradation pathway is suppressed under hypoxic conditions. This suppression of the alpha subunit allows dimerization with HIF-1β. The hypoxia response element (HRE) binds to the alpha subunit, which promotes the transcription of a cascade of genes that increase oxygen delivery, such as multiple angiogenic growth factors, cell metabolism, proliferation, and endothelial progenitor cell recruitment. Hence, HIF-1α is essential for angiogenesis, as it requires high expression levels in wounds under hypoxic conditions for wound healing. But in the case of diabetic wounds, despite having the hypoxic condition, the angiogenesis is inhibited due to impaired function and stability of hypoxia-inducible factor-1α (HIF-1α) as a diabetic wound environment poses hyperglycemia and increased reactive oxygen species (ROS), leading to a reduced formation of new blood vessels [20]. Factors like ROS and hyperglycemia also trigger antiangiogenic protein factors such as Thrombospondin-1 (TSP-1), Angiostatin, Interleukin-12 (IL-12), and Transforming Growth factor-beta (TGF-β) to be upregulated, and downregulation of the capillary maturation factors like Vascular Endothelial Growth Factor (VEGF) and Angiopoietin-1 (Ang-1). This downregulation of capillary maturation factors, in turn, reduces the migration of fibroblasts and keratinocytes [21]. This impaired migration of cells inhibits effective re-epithelialization, a crucial step in wound healing. Furthermore, an inadequate production of ECM due to the impaired migration of fibroblasts exacerbates the issue of wound closure [14,16,22].

### 2.4. Remodeling Phase

In normal wound healing, the newly formed tissue matures, strengthened by excess collagen degradation, creating wound contraction and resulting in scar formation [10,23] and rearrangement. This phase might extend for months or years and is active seven days after the injury. In this phase, Collagen III is replaced with collagen I, resulting in scar formation and mature scar tissue known as granulation tissue; the resulting scar from the collagen and ECM is produced by fibroblasts [5,16,24]. Studies on diabetes have shown that scars that recover have an altered shape and reduced collagen synthesis compared to scars formed by normal wounds. Both alterations lead to a scar with decreased tensile strength and increased collagen density, which hinders the proper healing of wounds. The scar also has a reduced capacity to contract [25]. The impaired functionality of fibroblasts in diabetic patients significantly hinders wound closure. In diabetic wounds, fibroblasts often become senescent and resistant to proliferation, further contributing to the healing impairment. Diabetic wounds that lack sufficient contraction rely more on granulation and re-epithelialization to heal, which makes the diabetic scar matrix less resilient to shear stress and tensile stresses [26]. It is speculated that the disrupted signaling pathways or altered cellular receptor activity cause the fibroblasts to be unresponsive to transforming growth factor-beta (TGF-β) and enhance aberrant ECM production. This fibroblast dysfunction impairs wound remodeling, prolonging healing and increasing complications [14,27].

## 3. The Complex Microenvironment of DFU

**Figure 2:**
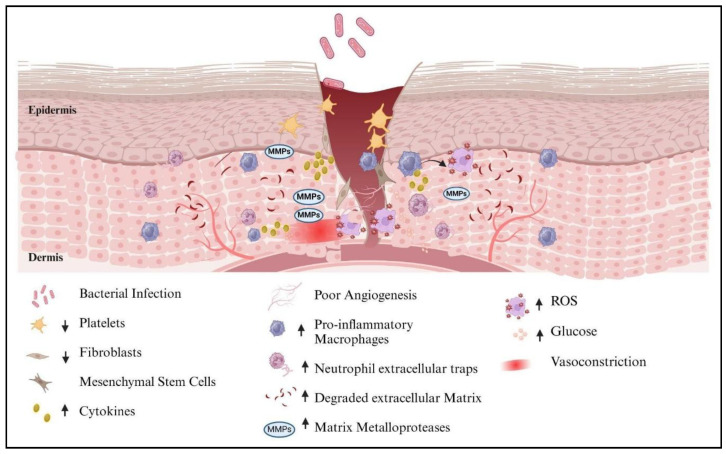
The microenvironment of Diabetic Foot (Image source: Created with BioRender.com).

The microenvironment of DFU is significantly complex. Figure 2 describes the extracellular matrix containing the cells and signals (such as growth factors) necessary for the healing of DFU. However, the existing clinical treatments for DFU are inadequate, prompting researchers to redirect their focus toward advanced techniques to overcome therapeutic obstacles associated with DFU [5]. The characteristics of the DFU microenvironment are discussed as follows:

Hyperglycemia is a defining characteristic of DFU and is closely associated with disruptions in insulin function, either through deficiency or resistance. In healthy conditions, intracellular glucose undergoes an oxidative metabolic pathway, producing ATP [5]. However, in hyperglycemic conditions, the polyol pathway becomes more active through increased glucose flux, making excess sorbitol using NADPH as a cofactor. As a consequence of NADPH reduction, glutathione production is affected, which leads to an increase in oxidative stress. The activation of the hexosamine pathway and protein kinase C pathway leads to insulin resistance. For instance, the formation of advanced glycosylation end products (AGEs) directly stimulates immune cells to produce high levels of reactive oxygen species (ROS), leading to increased oxidative stress, disruption of cell redox balance as a result of reduced activity of the antioxidant enzymes glutathione peroxidase and superoxide dismutase [28] and exacerbation of metabolic disorders in the wound area. Increased reactive oxygen species (ROS) can specifically harm peripheral nerve anatomy, metabolism, and blood flow [29]. It has been identified that hyperglycemia is a potential cause of dysfunction of endothelial cells by activation of various ROS-producing pathways and increased oxidant production in endothelial cells [30]. These endothelial cells are critical in wound healing in DFU as they show pressure-induced vasodilation, a normal response in skin protection [31].

Hyperinflammation in the context of DFU is closely linked to an imbalance in the regulation of redox homeostasis, which is induced by elevated blood sugar levels, as discussed previously. Diabetic foot wounds that do not heal are linked to elevated levels of the cytokines: IL-1β, TNF-α, and monocyte chemoattractant protein-1 (MCP-1) [32]. This delay in recruitment impairs the timely removal of neutrophils from the wound, resulting in an accumulation of oxidative stress. Moreover, this prolonged oxidative stress further contributes to the deterioration of fibroblasts, endothelial cells, keratinocytes, and mesenchymal stem cells, all of which play crucial roles in the healing process. Consequently, the formation of essential components such as granulation tissue, blood vessels, and epithelium is hindered. MMP-12 and other MMPs also cleave elastin and other ECM components, thereby sustaining the inflammation [33].

Hypoxic condition is a consequence of hyperglycemia. Hypoxia-inducible factor-1 (HIF-1) plays a major role in maintaining variation in hypoxic conditions. HIF-1 gets stabilized in response to local hypoxia during normal wound healing. HIF-1 regulates several cellular processes and stimulates the production of several angiogenic factors, including fibroblast growth factor-2, angiopoietin 2, and vascular endothelial growth factor (VEGF), to promote angiogenesis [34]. However, in diabetic conditions, increased glycemic conditions and oxidative stress can disrupt the normal regulation of HIF-1α. High levels of reactive oxygen species (ROS) can lead to oxidation of HIF-1α, affecting its stability and functionality. Diabetes often leads to impaired blood flow and reduced oxygen delivery to tissues, complicating the HIF-1α response. While HIF-1α should accumulate in response to hypoxia, its functionality may be compromised by inadequate oxygenation [35]. To promote wound healing in patients with DFU and to decrease the amputation risk, hyperbaric oxygen therapy is used in clinical settings. However, it is important to note that the treatment is only affordable for a small population. Therefore, it is important to develop novel methods for treating DFUs [36].

In normal wound healing, the pH of the wound environment is typically acidic, with a range of pH 4 to 6 [37]. Hunt and Beckert studied the presence of necrotic and devitalized tissue in the wound, which increases the wound’s metabolic load and leads to tissue hypoxia. Chronic recurring wounds, such as venous leg ulcers, cause permanent barriers to oxygen delivery due to atrophic and scarred skin and local vasculature. Hence, oxygen deficiency in the wound area is one of the major factors that causes the alteration of pH [38]. In normal wounds, the skin surface maintains an acidic pH environment as oxyhemoglobin releases oxygen, while reduced hemoglobin carries a net negative charge that binds hydrogen ions, supporting a protein buffer system within tissue and blood. This acidic microenvironment is crucial for wound healing, as it regulates enzymatic activity, collagen biosynthesis, and immune responses [39]. Supporting this, a study demonstrated that externally maintaining wounds at pH 4 significantly improved healing in vivo. Mice treated with pH 4 acid buffers showed accelerated re-epithelialization, enhanced collagen deposition, and smaller wound areas by day 7 compared to controls [37]. However, in diabetic wounds, tissue necrosis and hyperglycemia impair oxygen delivery, making it difficult to maintain acidic pH. As a result, the wound environment tends to shift toward alkalinity, with pH levels often ranging from 7.15 to 8.9. Reduced oxygen tension contributes to these pH changes, negatively affecting collagen synthesis, immune responses, and angiogenesis [40]. In a study, Schultz and Mast reported that the deamination of amino acids by wound-colonizing bacteria contributes to an alkaline microenvironment. The production of ammonia and other alkaline byproducts from bacterial metabolism significantly alters wound pH [41]. This dynamic pH progression holds significance in understanding the intricacies of wound healing and its implications for diabetic patients [36].

Bacterial infection represents a significant complication in the context of chronic wounds. The wound is highly prone to infection during the healing process, with *Staphylococcus aureus* and *Pseudomonas aeruginosa* being the most frequently encountered pathogens. *Enterococcus, anaerobes*, and gram-negative bacteria, such as *Escherichia coli*, *Klebsiella* species, and *Proteus* species, have been identified in diabetic wound infections [42]. Wound colonization becomes more severe when bacteria form biofilms; these different microorganisms create micro-communities within a matrix of extracellular polymeric substances called biofilms. This matrix serves to provide additional protection and attachment. Several chronic infections are primarily caused by these biofilms, which also facilitate the reemergence of multidrug-resistant strains and treatment failure [43]. In DFU, the biggest challenge is the increased susceptibility to several possible infections that may result in grave consequences like infection, gangrene, osteomyelitis, amputation, or even death [44]. In DFU, individuals with infections have a 50% higher risk of amputation [45]. Moreover, host immune cells also struggle to effectively infiltrate these biofilms, thereby making the eradication of bacteria a notably arduous task [46].

The MMPs are members of a zinc-containing proteolytic enzyme family, and their function in the breakdown of extracellular matrix was initially identified in tadpoles [47]. One characteristic of DFUs is the overexpression of MMPs, which prolongs Inflammation. MMPs that are overexpressed in DFUs might lead to severe tissue deterioration and poor wound healing. Because increased MMPs disrupt the extracellular matrix and obstruct growth factors, which are both necessary for wound healing, reduced wound healing is believed to result from elevated levels of MMPs [48]. Contrastingly, not all MMPs are destructive in DFU. For instance, the MMPs with beneficial effects, MMP-1 and MMP-8, are downregulated, and MMPs that have detrimental wound healing effects, MMP2 and MMP9, are upregulated [33,49]. Some MMPs, such as stromelysins (MMP-3 and 10) and membrane-type MMPs (MMP-14 and 19), and their significance remains unclear [50].

This review mainly focused on a prominent complication of Diabetes Mellitus, namely, Diabetic foot ulcer. The existing approaches for its treatment explore the necessity for a novel therapeutic intervention and center our focus on the utilization of hydrogels as a potential treatment modality for DFU.

## 4. Hydrogels

Hydrogels have the capacity to maintain the crosslinking in a swollen state. The swelling nature of hydrogel is due to the presence of polar hydrophilic moieties such as -OH, -SO_3_H, -NH_2_, -COOH, -CONH_2,_ etc., along the polymer network as branched groups. The water holding capacity helps the hydrogels have a degree of elasticity close to that of natural tissue, enabling them to mimic the natural conditions of tissues in cell cultures. Hydrogels offer distinct advantages over traditional wound dressings, particularly for treating dry or mildly exudative wounds like DFUs.

A systematic review and meta-analysis by Saco et al. compared five dressing types: (1) Alginates, a highly absorbent wound dressing material developed using alginate polymer, mainly used for wounds to absorb the exudate. (2) Foams are soft, spongy, absorbent materials that help in the absorption of wound exudates and cushioning the wound (e.g., polyurethane, silicone-based). (3) Hydrocolloids are opaque, translucent bandage-like materials containing a gel-forming agent such as CMC in the form of film, powder, or spray foam (e.g., CMC, gelatin, and pectin-based), (4) hydrofibers are soft, highly absorbent material that forms a gel when it comes in contact with wound exudate (e.g., chitosan, gelatin, HA-based), and (5) hydrogels, and found that only hydrogels demonstrated a statistically significant improvement in full ulcer healing with a risk ratio (RR) of 1.80 (95% Confident interval (CI), 1.27–2.56) over basic wound contact dressings. Despite some uncertainty regarding bias, this finding led to a grade 2A recommendation in favor of hydrogels, supported by moderate-quality level B evidence from three randomized controlled trials [51]. Since hydrocolloid dressings are occlusive, maintaining a hypoxic environment leads to the liquefaction of necrotic tissue and aids autolytic debridement. Case studies reporting the effectiveness of hydrocolloid dressings in facilitating autolytic debridement of DFUs have reinforced this hypothesis [52]. Unlike hydrocolloids, foams, and alginates, which did not show significant differences in healing efficacy, hydrogels effectively maintain an optimal moisture balance, facilitating cellular migration and autolytic debridement. Their non-adherent nature prevents trauma during dressing changes, a drawback associated with hydrocolloids that can adhere tightly to the wound. While foams enhance exudate absorption, excessive drying may slow healing, and alginates, though highly absorbent, are more suited for heavily exuding wounds rather than DFUs, which typically produce moderate exudate [51]. Even though the Foam dressings attempt to rectify the lack of exudate absorbency of occlusive dressings, without compromising on the moist environment needed for tissue repair [53].

A systematic review of randomized controlled trials (RCTs) by Dumville et al. found no statistically significant difference in healing rates between foam dressing and basic wound dressing materials for DFU treatment, with a reported relative risk (RR) of 2.03 (95% CI: 0.91 to 4.55). Similarly, when foam dressing was compared with alginate dressings, the findings again showed no significant difference in healing outcomes, RR 1.50 (95% CI: 0.92 to 2.44). Additionally, a separate study within the same review involving 40 participants compared foam dressings with hydrocolloid dressings and also reported no statistically significant difference in the number of DFUs healed (RR 0.88, 95% CI: 0.61 to 1.26). These collective findings suggest that foam dressings do not demonstrate a consistent therapeutic benefit over commonly used dressing types and may not be considered a reliable choice for promoting DFU healing [54]. By actively hydrating the wound bed rather than relying solely on absorption, hydrogels create a favorable healing environment while reducing the risk of peri-wound maceration. Their inherent cooling effect also helps alleviate discomfort, making them a more patient-friendly option. Despite concerns regarding variations in fluid-handling capacity among other dressing materials, clinical data indicate no significant differences in their efficacy [51]. Due to their capacity to maintain moisture balance without excessive absorption or adhesion, hydrogels are considered a versatile and effective choice for DFU management [55]. Additionally, bioadhesive, antimicrobial, and regenerative properties of hydrogels make them highly effective in wound dressing applications. Moisture balance of hydrogel promotes re-epithelialization and guarantees ideal wound hydration and hemostasis. Hydrogels can be tailored for cell adhesion, degradability, and viscoelasticity, which affects cell differentiation and proliferation. By controlling immune cell activity and releasing cytokines, hydrogels also improve immune responses. The gas exchange made possible by their porous structure promotes the healing of DFUs [51]. In contrast to other biomaterials, hydrogels are advantageous due to a porous structure, high biocompatibility, tunable mechanical properties, biodegradability, and non-toxicity. Their numerous applications in drug delivery, tissue engineering, biosensors, wound dressing materials, and self-healing biomaterial-based hydrogels have gained attention [56,57].

Hydrogels are classified as natural, synthetic, and composite based on their sources. Natural polymers are materials that are widely available in nature or polymers that can be extracted from plants, animals, fungi, or bacteria. These materials are used in the development of natural polymer hydrogels. In contrast, synthetic polymers are artificially synthesized in laboratories. By using these materials, synthetic polymer hydrogels can be developed. Composite hydrogels are combinations of these natural and synthetic polymers cross-linked with each other. Considering the classification based on their source is essential, as the polymers used in the construction of hydrogels can have a significant impact on controlling toxicity, biocompatibility, and mechanical properties. A detailed review of these hydrogels and their advantages is discussed below.

### 4.1. Types of Hydrogels for DFU

#### 4.1.1. Natural Polymer Hydrogels for DFU

Natural polymers include polysaccharides, polypeptides, and polynucleotides. These polymers are abundant in nature, inexpensive, non-toxic, inert, and biodegradable. Additionally, functional groups such as -OH, -COOH, -COO, etc., in these polymers make cross-linking more efficient. These polymers show well-defined, covalently bonded monomeric units with a larger structure, which facilitates the development of targeted and controlled drug delivery systems. Natural polymers are generally high molecular weight compounds with linear, long chains of repeating units of monomers that are widely present in animals, plants, and other organisms in the form of proteins, polypeptides, chitin, alginate, starch, agar, cellulose, and so on [58]. Such polymers are used as base materials alone or combined with other materials. For instance, the behavior and mechanism of cellulose-chitosan hydrogels have been demonstrated by Li and Bai [59]. Some cellulose derivatives, such as methylcellulose, hydroxymethyl cellulose, carboxymethyl cellulose, etc., have been utilized to develop hydrogels. The derivative forms of cellulose, containing methyl, hydroxy, or other functional groups, help control cross-linking, hydrophilicity, etc. [60]. Kumar et al. developed a hydrogel using hydroxypropyl methylcellulose and collagen composite scaffolds fabricated with a povidone-iodine model drug for wound healing. The proliferation efficiency of the hydrogel was proved on NIH3T3 fibroblast cells without exhibiting toxicity. This study showed that natural polymers like cellulose derivatives and collagen could be effective in tissue regeneration without toxicity [61]. A recent study reported that incorporating systemic melatonin with a bacterial nanocellulose hydrogel significantly improved the repair of full-thickness skin wounds in diabetic rats. By the 14th day, wounds treated with this combination showed almost complete closure, accompanied by enhanced fibroblast proliferation and diminished inflammatory response compared to untreated groups [62]. Chitosan-based hydrogels are widely studied polymers. Chitosan is a derivative of chitin, which is a natural, amino-homogeneous linear polysaccharide. Stimuli-responsive intelligent hydrogels from chitosan have been developed to demonstrate the promotion of wound healing [63]. Cyclodextrins are another class of polysaccharides, cyclic oligosaccharides produced from starch and its derivatives. These can entrap other molecules in their lipophilic core via supramolecular complex formation. This enables the potential application of cyclodextrin in developing drug-delivery systems to treat chronic wounds [64]. Shu et al. constructed fiber-reinforced gelatin (GEL)/β-cyclodextrin (β-CD) therapeutic hydrogel incorporated with platelet-rich plasma exosomes (PRP-EXOs) to improve diabetic wound healing. In diabetic rat models, these hydrogels provided a biocompatible environment for cell adhesion, proliferation, and tissue regeneration. PRP-EXOs activated autophagy and inhibited apoptosis in endothelial cells and fibroblasts, promoting blood vessel formation, collagen synthesis, and re-epithelialization [65].

Self-healing hydrogels are pioneering advancements in the field of hydrogels in the present decade. The cyclodextrins showed promising results in developing self-healing hydrogels. Supramolecular complex-based self-healing hydrogels fabricated using cyclodextrins have been illustrated by Yong-Guang [66]. The structural diversity of natural polymers helps form cross-links and provides biocompatibility by resembling the components of biological extracellular matrices; hence, natural polymers are readily acceptable to the body and show more bioactivity. All these factors provide factual information that natural polymers are suitable for research. Natural polymer-based hydrogels generally exhibit weaker mechanical strength. For instance, collagen-based hydrogels typically have a Young’s modulus in the range of 0.001 to 0.01 MPa. Chitosan hydrogels exhibit values of approximately 0.01 to 0.5 MPa, and carrageenan hydrogels fall within 0.001 to 0.1 MPa [67]. Although mechanical properties are still challenging when natural polymers are selected to develop hydrogels, several attempts have been made to improve mechanical strength, such as introducing a dual network between polymers [68]. Synthetic polymers were introduced to develop hydrogels to address the limitations posed by weak mechanical properties.

#### 4.1.2. Synthetic Polymer Hydrogels for DFU

Synthetic polymers are formed by the chemical reactions of organic molecules. These polymers have highly tunable chemical and physical properties compared to natural polymers and can be synthesized with an extended polymeric chain of a high molecular weight. So far, synthetic polymers such as PEG, PVA, poly (acrylic acid) (PAA), poly (2-hydroxyethyl methacrylate (PHEMA), poly (acrylamide) (PAAm), and poly (ethylene oxide) (PEO) have been studied. Poly (acrylamide) hydrogels have been synthesized and used in many fields. These hydrogels possess a hydrophilic nature and show distinct physical and chemical properties that can be utilized to immobilize cells and enzymes, drug-delivery systems, etc. Hina et al. demonstrated self-healing poly (acrylamide) clay hydrogels cross-linked via free radical reaction. The developed hydrogels showed strong mechanical robustness up to 0.4 MPa tensile strength and hydrolytic stability [69]. Synthetic polymer hydrogels are particularly useful for developing high-strength materials. For instance, double network hydrogels composed of poly(acrylic acid) (PAAc) and poly(N-isopropyl acrylamide) (PNIPAm) have demonstrated remarkable mechanical properties, achieving tensile strengths in the range of 2 to 4.6 MPa [70]. Few studies showed that copolymerization of acrylamide with other polymers will increase the mechanical strength of acrylamide-based hydrogels. Nesrinne et al. synthesized poly (acrylamide-co-acrylic acid) based hydrogel using a free radical polymerization mechanism. Their rheological studies showed increased mechanical strength of cross-linked polymers [71]. Synthetic polymers are advantageous in developing stimuli-responsive hydrogels. Stimuli like temperature and pH will trigger the response of hydrogels. This enables the development of targeted and controlled drug delivery to targeted sites. Poly (N-isopropylacrylamide) (PNIPAM) is one of the important thermosensitive polymers. The effect of temperature on PNIPAM-based hydrogels has been studied, with findings demonstrating that the elasticity of PNIPAM hydrogels is temperature-dependent [72]. A study showed the combination of mesoporous silica and PNIPAM in creating composite hydrogels for efficient drug loading and release [73]. Certain synthetic polymers, such as polyacrylamides, exhibit slow degradation, and their residual or degraded monomers have been known to be cytotoxic [74]. Despite having certain advantages, they also have drawbacks such as cytotoxicity, poor biocompatibility, poor bioactivity, poor environmental degradability, and cost, which restrict their use in biomedical applications.

#### 4.1.3. Composite-Polymer Hydrogels

These hydrogels are typically composed of combinations of natural and synthetic polymers or, in some cases, ceramic components such as nanoclays. Generally, two different polymers are crosslinked to form an interpenetrating mesh-in-mesh network [75], while nanomaterials such as particles or composites are incorporated in a heterophasic manner, acting as reinforcing fillers within the hydrogel matrix [76]. Natural polymers show mechanically weak properties. In contrast, synthetic polymers have excellent mechanical strength, but their toxicity limits their application. To overcome these problems, some researchers aimed to fabricate hydrogels by combining these polymers. The studies demonstrated the retention of stimuli-responsive properties while exhibiting reduced toxicity compared to synthetic polymer-based hydrogels. For instance, Stimuli-sensitive chitosan grafted with poly (acrylic acid), poly (hydroxypropyl methacrylate), PVA, and gelatin hydrogels were synthesized using gamma rays [73]. Mechanically responsive smart hydrogels have been developed using lignin and lignin-based derivatives. Lignin, a natural polysaccharide abundant in nature, is easily available, biodegradable, non-immunogenic, and cost-effective. It was crosslinked with another natural polymer, beta-cyclodextrin, and a synthetic polymer, poly (ethylene glycol) methyl acrylate (PEGMA). This composite overcomes challenges such as cytotoxicity and biocompatibility due to PEGMA as its synthetic component. Also, it helps overcome challenges of natural polymers, such as mechanical properties, and induces stimulus responsiveness, making safe biomedical uses of this hydrogel [77]. Zhou et al. prepared a semi-IPN of carboxyethyl chitosan and PHEMA hydrogels with pH-responsivity through photopolymerization, which demonstrated good mechanical strength and sustained drug release [78].

The concept of composite hydrogels also involves nanotechnology. Nanocomposites, nanosponges, nanogels, and many other forms of nanoparticles, when combined with polymers as a base material, showed significant increases in the functionality of the hydrogels, such as increased modulus, tunable viscoelasticity, stimulus sensitivity, drug release, and increased drug loading capacity. Mahmoudian and Ganaji have studied chitosan-glycerophosphate-based hydrogels loaded with vancomycin hydrochloride HMPC nanoparticles. The noticeable effect of prolonged drug release was observed due to the diffusion of the transport mechanism [79]. Composite hydrogels are heterogeneous, and this helps in cell-cell interaction, cell organization, and adhesion for medical applications. Hence, these hydrogels have good potential as chronic wound patches [80]. Drug-loaded hydrogels provide controlled drug release, maintaining optimal concentrations of antibiotics, antimicrobial peptides, and growth factors that enhance local infection control while minimizing systemic side effects. Another investigation engineered a sodium alginate hydrogel infused with disulfiram (DSF) (DSF-NPs@SA) to facilitate the healing of diabetic wounds through the modulation of inflammatory responses and the enhancement of tissue regeneration. In a mouse model simulating diabetic wounds, the DSF-NPs@SA formulation markedly expedited the process of wound closure, achieving complete healing by the fifteenth day, in contrast to the protracted recovery observed in control cohorts. The hydrogel reduced neutrophil extracellular trap (NET) formation, inhibited the Caspase-1/GSDMD signaling pathway, and facilitated the polarization of M2 macrophages, thus alleviating chronic inflammatory conditions. Furthermore, it promoted collagen deposition, neovascularization, and re-epithelialization, collectively contributing to expedited tissue repair. These findings highlight the promising potential of drug-encapsulated hydrogels as efficacious wound dressings, providing controlled drug release and targeted therapeutic intervention [81]. These evidential studies demonstrate that composite hydrogels help develop hydrogels with desirable properties and provide promising advancements in medicine, such as wound healing patches. Table 2 provides a comparative overview of natural, synthetic, and composite hydrogels used in the treatment of DFUs, highlighting their respective advantages and disadvantages.

### 4.2. Self-Healing Hydrogels

Self-healing hydrogels are a new class of hydrogels that are designed to autonomously repair themselves after mechanical damage. This property helps restore mechanical properties, thereby helping maintain structural and functional integrity over time. This self-healing capability is achieved through dynamic, reversible bonds within the hydrogel network, such as Hydrogen bonds, Ionic interactions, and dynamic covalent cross-linking applications [87]. These hydrogels can adapt to the mechanical stress and deformation often encountered in wound sites, ensuring continuous protection and optimal healing conditions. The self-healing property is particularly beneficial in wound healing applications as it ensures prolonged and adequate wound coverage, minimizing the need for frequent dressing changes, which can disrupt the healing process and introduce potential infection. Generally, hydrogels are capable of adapting to the wound bed, while their self-healing properties help maintain structural integrity when mechanical damage occurs due to movements of joints, such as bending, straightening, and rotational motions, thereby ensuring close contact with the tissue and enhancing the delivery of therapeutic agents. A study conducted by Liang et al. showed that dual-dynamic-bond cross-linked hydrogels combining catechol-Fe coordinate bonds and Schiff base bonds enable self-healing and on-demand removal while facilitating the release of bioactive Qatarized Chitosan, a modified chitosan with antibacterial properties that effectively kills (100% killing) bacteria like MRSA, a commonly present bacterial pathogen in DFU infections. On the other hand, its NIR (near-infrared) responsiveness promotes photothermal ablation of bacteria, and the hydrogel showed rapid self-healing within 2 min. The photothermal performance of the hydrogel was approximately 50 °C within 5 min, which is sufficient for effective photothermal antibacterial action without damaging the surrounding tissue. MRSA-infected full-thickness skin wounds in rats showed that the hydrogel-treated group achieved complete wound closure in 14 days compared to 21 days in the untreated control group, demonstrating significant acceleration in wound healing [88]. This system ensures sustained delivery of bioactive materials, accelerating wound closure.

### 4.3. Stimuli-Responsive Hydrogels

**Figure 3:**
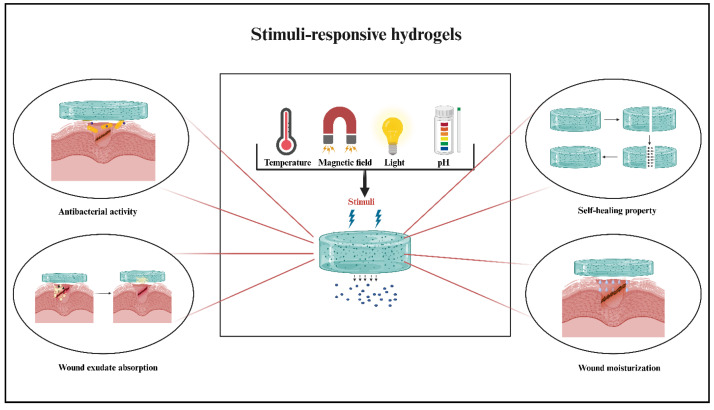
Stimuli-responsive hydrogel responsive to pH, temperature, and light, designed for targeted drug delivery and wound management.

Hydrogels with stimulus-dependent functionality, known as smart or stimulus-responsive hydrogels, are engineered to exhibit stimulus-responsiveness and self-healing properties. Polymers, including natural and synthetic, such as Poly (N-isopropylacrylamide) (PNIPAM), poloxamers, PEG, PLA, Chitosan, Thiol-modified hyaluronic acid, gelatin, and fibrin, are used in developing pH, temperature-responsive hydrogels. These hydrogels can show a switchable sol-gel transition when external triggers are applied. Mainly, stimuli fall into two categories: endogenous and external. The endogenous stimuli are organism-specific and involve variations in temperature, redox gradient, pH, and enzyme concentration. Exogenous stimuli, including light, ultrasound, magnetic or electric fields, and other artificial stimuli, are administered outside the body [89]. In contrast to conventional hydrogels, stimuli-responsive hydrogels facilitate the spatially controlled release of bioactive molecules at the wound sites, including antimicrobials, hemostatic drugs, anti-inflammatory molecules, and epidermal growth factors, which could speed up the healing process [90]. A few major stimuli-responsive hydrogels are shown in Figure 3 and discussed in this article. The advanced functionality of stimuli-responsive hydrogels exhibits self-healing properties and reacts dynamically to external cues such as pH shifts, temperature fluctuations, magnetic fields, and light irradiation. These hydrogels leverage reversible physical or chemical interactions, such as hydrogen bonding, ionic interactions, and covalent cross-links, to modulate their structural and mechanical properties in response to environmental changes [91].

#### 4.3.1. Thermo-Responsive Hydrogels

Thermal-responsive hydrogels are widely studied in stimuli-based hydrogels. Usually, hydrogels can be synthesized using natural or synthetic polymers, which may contain hydrophobic functional groups. These functional groups help in creating hydrogen bonds between other polymeric molecules. Some groups, like methyl, ethyl, propyl groups, etc., facilitate physical and conformational changes with temperature changes. Thermo-responsive hydrogels are based on the principle that hydrophilic functional groups in the system form hydrogen bonds with hydrophilic functional groups in the surrounding system when the temperature falls below the lower critical solution temperature (LCST). Below the LCST, the hydrophilic groups in the polymers, such as amide groups of poly(N-isopropyl) acrylamide, PNIPAM, form hydrogen bonds with water molecules, making the hydrogel soluble. This improves the solubility of hydrogels and allows them to be injected in situ as a solution. As the temperature rises above the LCST, the hydrophobic interactions between the polymer chains, such as isopropyl groups in the PNIPAM, become dominant, leading to the collapse of the hydrogel network and the expulsion of water. This results in a gel-to-solid transition. The gel-to-solid phase transition is enabled in thermo-responsive hydrogels [92]. This transition does not need any assistance or triggers, such as enzymes. In developing these types of hydrogels, toxic cross-linking agents can be avoided. Hence, thermogels show intrinsic biocompatibility as an injectable in-situ hydrogel [93]. Some synthetic polymers, such as Poly(ethylene glycol), poly (propylene glycol), poly(vinyl alcohol), and poly(N-isopropyl acrylamide), have been used extensively. PNIPAM is the most commonly researched thermogel polymer with LCST properties. PNIPAM is a homopolymer with low cytotoxicity and an LCST of around 32 °C. PNIPAM is typically copolymerized with other hydrophilic polymers, such as chitosan and others, to increase the LCST and thus can be suitable for physiological thermo-responsiveness. Its phase separation behavior has been widely studied [94]. Pluronics^®^ or poloxamers, specifically Pluronic F-127 (PF127), exhibit sol-gel transition near 37 °C, making them ideal for drug delivery applications due to their unique thermo-responsive properties [95]. These are advantageous over conventional hydrogels as the temperature-dependent swelling and deswelling enable the controlled release of therapeutic agents. The thermoresponsive hydrogel holds promising results in addressing various challenges in the wound care sector.

#### 4.3.2. pH-Responsive Hydrogels

The pH changes can trigger medication release in several bodily locations, including the blood vessels, gastrointestinal tract, and vagina. pH-responsive hydrogels are designed to swell or deswell in response to changes in pH, which is particularly useful for targeting specific physiological environments, such as the acidic pH of infected wounds or the alkaline pH of chronic wounds. Local pH changes can be used to modulate the release of drugs based on specific substrates. pH-responsive hydrogels are generally constructed using polymeric backbones with ionic pendant groups such as carboxyl or amino groups that ionize in response to pH changes. For example, in polymers such as Poly (acrylic acid) and Poly (methacrylamide), carboxyl groups (-COOH) remain protonated in an acidic environment, leading to reduced electrostatic repulsion and hydrogel shrinkage. In an alkaline environment, carboxyl groups deprotonate to form carboxylate anions (COO-), increasing electrostatic repulsion, which causes the hydrogel to swell. The electrostatic repulsive forces generated are responsible for pH-dependent swelling and hydrogel deswelling, thereby helping achieve controlled drug release [96] in the hydrogel mesh network.

Additionally, pH-sensitive polymeric networks have been used to administer insulin. In this case, a saturated insulin solution was enclosed within a pH-responsive hydrogel made of hydroxyethyl methacrylate-based copolymer, in which the enzymes catalase and glucose oxidase were immobilized. When glucose diffuses into the hydrogel, glucose is converted to gluconic acid via an enzyme-catalyzed process that lowers the pH in the hydrogel microenvironment. Thus, it allows the pH-responsive swelling of the hydrogel. The insulin delivery rate is reduced when the hydrogel contracts in response to a drop in the glucose concentration, which is triggered by the released insulin. Drug release was able to satisfy the physiological needs through a self-regulating drug delivery system [97]. A konjac glucomannan-based self-healing hydrogel was developed that showed pH-dependent sol-gel behavior. When pH was reduced, the hydrogel showed gelling in acidic pH and hardening when pH was increased by adding NaOH [98]. This pH-dependent behavior allows for controlled drug release.

In diabetic wounds, where the pH is often alkaline, the hydrogel can swell and release the therapeutic agents. For instance, in a study, researchers have developed a pH-responsive hydrogel that demonstrates pH-sensitive drug release using sodium alginate and chitosan cross-linked with calcium by incorporating a multifunctional nanozyme (Mo, Fe/Cu, I-Ag@GOx) system that enables cascade catalytic reactions that produce ROS to kill bacteria and later decompose excess ROS to generate oxygen, alleviating hypoxia and oxidative stress. The diabetic wound models showed significant enhancement in angiogenesis and tissue regeneration, with 92.3% wound closure by day 12. This also helped modulate infection, glucose levels, hypoxia, and oxidative damage. In the early phases of wound healing in DFU, the wound environment tends to be slightly acidic, with a pH of around 6.5. At pH 6.5, sodium-chitosan hydrogel destabilizes, leading to increased swelling and enhanced release of the embedded nanozyme (Mo, Fe/Cu, I-Ag@GOx). The drug release was higher than pH 7.4, aligning with acidic wound conditions in the early stages [99]. A pH-responsive hyaluronic acid-collagen hydrogel was developed to release metformin in acidic diabetic wound environments. This hydrogel promoted macrophage polarization from M1 to M2 phenotype, reducing inflammation and enhancing ECM remodeling. In diabetic mouse models, wounds treated with the hydrogel achieved 90% closure by day 14, compared to 63% in controls. M2 presence increased by 2.3-fold, and collagen formation under high-glucose conditions indicated enhanced tissue regeneration [100]. Hence, pH-responsive hydrogels have become a powerful tool in tissue engineering and drug delivery.

#### 4.3.3. Magnetic-Stimuli Responsive Hydrogel

Magnetic-responsive hydrogels are developed by incorporating molecules that show magnetic properties in a magnetic field. Magnetic nanoparticles, which can alter their structures and characteristics and work in response to magnetic fields, are added to crosslinked polymers to synthesize magnetic-responsive hydrogels. To generate magnetic hydrogels for biomedical applications, a variety of magnetic nanoparticles, such as iron oxide (Fe_3_O_4_, γ-Fe_2_O_3_) [101], transition metal alloys (FePt) [102], and transition metal ferrites such as CoFe_2_O_4_, MnFe_2_O_4_, etc., can be incorporated into the hydrogels as magnetic-responsive components. However, Fe_3_O_4_ nanoparticles have gained attention and are the most widely used in the biomedical and pharmaceutical sectors due to their advantages, which include high magnetization ability, good compatibility with tissue, and relative ease of preparation and functionalization [103]. The magnetic nanoparticles in the nanogel can self-assemble to form an ordered structure when exposed to a magnetic field. This property causes the surface of the hydrogel to be anisotropic, promoting the growth of new cells, neurogenesis, signal transmission, and extracellular matrix regeneration [104]. An alternating magnetic field (AMF) causes magnetic nanoparticles to oscillate or rotate, resulting in heat generation and an increase in temperature. Thus, it promotes drug release by enhancing the movement of drug molecules and the degradation of polymers. Furthermore, it can treat tumors in combination with chemotherapy [105].

The design and characteristics of external magnetic fields serve a crucial role in the ability of magnetic hydrogels to respond to external stimuli and exhibit various magnetic properties in biomedical applications. In a study, researchers developed a multifunctional Fe-Se-HA bilayer microneedle (MN) patch for treating chronic diabetic wounds. The sharp microneedles can pierce scabs and penetrate deep bacterial biofilms, delivering selenium nanoparticles (SeNPs) directly to infection sites. Activated by a custom Disk-ZVS electromagnetic field, the ferromagnetic tips convert magnetic energy into localized heat to kill bacteria without harming surrounding tissue. As the microneedles degrade, SeNPs are gradually released, reducing oxidative stress and stimulating angiogenesis. This system integrates mechanical penetration, magneto-thermal disinfection, anti-inflammation, and tissue regeneration, offering a precise and non-invasive therapy for deep, infected diabetic wounds [106]. In another study, a tannin-bridged magnetic responsive multifunctional hydrogel was developed using polyvinyl alcohol integrated with cobalt ferrite nanoparticles (CFO NPs), bridged by tannic acid (TA). Under an external static magnetic field (SMF), the CFO NPs induce deformation in the hydrogel, leading to surface topographical changes that promote cell adhesion and proliferation. Additionally, the hydrogel exhibits antibacterial and anti-inflammatory properties due to the synergistic effects of TA and CFO NPs. In vivo studies demonstrated that the hydrogel, when combined with SMF, significantly accelerated wound healing and promoted early vascularization, highlighting its potential for clinical applications in wound repair [107]. Magnetic-responsive hydrogels represent a promising platform for advanced wound care, offering precise, non-invasive control over therapeutic delivery, enhanced antibacterial efficacy, and mechanical stimulation. Their ability to respond dynamically to external magnetic fields allows for spatiotemporal regulation of healing processes, particularly beneficial in chronic and hard-to-heal wounds. However, challenges remain, including the potential long-term cytotoxicity of magnetic nanoparticles and the need for optimized device integration for clinical use.

#### 4.3.4. Photoresponsive Hydrogels

Photoresponsive hydrogels comprise a polymeric network with a photoreactive moiety, normally a photochromic chromophore. It undergoes physical and chemical changes in response to an optical signal called smart hydrogels. Functional groups are essential for modifying hydrogels’ chemical, physical, and biological characteristics and generating diverse reactions. Incorporating photoreactive moieties such as Thiol-ene, trithiocarbonate, disulfide, alkyl sulfide, and azobenzene into hydrogels can facilitate substrate release, shrinkage, cross-linking, photothermal excitation, and reactive site activation [108]. The physicochemical characteristics of these chromophores, such as their dipole moment and geometric structure, as well as the macroscopic hydrogel’s shape, structure, and characteristics, are altered due to swelling and contraction due to their photosensitivity. Typically, these hydrogels can be synthesized by chemical-based polymer cross-linking methods, such as host-guest interactions, free radical polymerization, and metal-ligand complexation. Based on the photosensitive molecules used in incorporation, these molecules exhibit molecular mechanisms such as photocleavage, photoisomerization, and photodimerization. Polymers such as PAAm, PAA, β-Cyclodextrin, PEG, and acrylamide are used in developing photosensitive hydrogels. Photoresponsive molecules such as azo-benzene have been used as photoresponsive molecules [109].

Utilizing a photosensitive hydrogel as a drug carrier can lead to a successful therapeutic outcome. For example, a photosensitive nanogel was developed by Patnaik et al. using azobenzene-modified dextran, in which the hydrophobic azobenzene along the side chains merged with the hydrophilic backbone. The isomerization of the azobenzene groups under UV radiation caused the hydrophobic contacts to diminish, which sped up aspirin escape from the matrix [110]. Chen et al. developed a strategy for covalent tethering a photoresponsive spiropyran layer (≈1.2 µm) to a hydrogel surface via quaternization reactions of tertiary amino groups with an iodide-functionalized spiropyran [111]. The developed hydrogel surface was superhydrophobic, which was attributed to both the hydrophobicity of spiropyran and the formed hierarchical micro/nanoscale surface roughness. When the spiropyran-coated hydrogel was irradiated with UV light, the surface switched from superhydrophobic to hydrophilic, thus allowing photo-controlled diffusion of polar substrates in or out of the hydrogel, which was demonstrated for the inhibited release of fluorescein from a hydrogel in water. Xiaoliang et al. showed the controlled drug release using UV that activates the prodrug and 808 nm near-infrared light that enhances the catalysis of nanoparticle indole-3acetic acid/zeolitic imidazolate framework-8@polydopamine@platinum (IZPP). In this study, researchers have developed a silk fibroin-based hydrogel embedded with an ATP-activated prodrug system (ISD3) consisting of nanoparticles IZPP. This hydrogel targets multidrug-resistant bacteria-infected pressure ulcers. These studies show a promising medical application, particularly in photothermal and cancer therapy. However, further research is necessary to optimize its photoresponsiveness and scalability for practical implementation [112].

Another preclinical study conducted by Kang et al. utilized living Hematococcus (HEA) hydrogels, a distinctive variety of microalgae-infused hydrogel characterized by its high efficiency in oxygen generation, alongside Astaxanthin (AST), a potent antioxidant pigment known for its robust reactive oxygen species (ROS) scavenging and anti-inflammatory attributes, demonstrating efficacy in the healing of diabetic wounds through a multifunctional, light-responsive paradigm. The application of high-intensity light (658 nm, 0.5 W/cm^2^) facilitates the photothermal antibacterial properties of GHEA@Gel, thereby assisting in infection control. Conversely, low-intensity light promotes oxygen production, alleviating hypoxia and encouraging vascularization. In vitro studies indicate that HEA@Gel enhances cellular proliferation, migration, and vascularization, which collectively support the process of tissue regeneration. In vivo investigations reveal that HEA hydrogels expedite the closure of wounds, mitigate inflammation, and enhance tissue remodeling in DFU [113]. An innovative immunomodulatory hydrogel (GHM3) for the treatment of diabetic DFUs by simultaneously addressing hyperglycemia-induced inflammation and excessive reactive oxygen species (ROS). AuPt nanoparticles in the hydrogel absorb near-infrared (NIR) light, upon NIR irradiation, generate localized heat (hyperthermia) due to their plasmonic properties. This heat enhances enzymatic and catalytic reactions, promoting glucose depletion. Additionally, AuPt nanoparticles mimic glucose oxidase-like activity, catalyzing glucose oxidation into gluconic acid and hydrogen peroxide. The generated hydrogen peroxide is further decomposed by the hydrogel’s melanin component, thereby reducing oxidative stress. Utilizing AuPt@melanin nanocomposites, the hydrogel enables hyperthermia-assisted glucose depletion and ROS scavenging, effectively disrupting the ROS inflammation cycle. In vitro and in vivo studies demonstrate that GHM3 promotes M1-to-M2 macrophage polarization, enhancing wound healing in diabetic rats. This approach offers a facile, safe, and efficient therapeutic strategy for DFUs, with potential implications for advanced diabetic wound management [114].

## 5. Hydrogels for DFU Treatment Targeting Phases of Wound Healing

Effective treatment strategies must address the distinct phases of wound healing, namely, hemostasis, inflammation, proliferation, and remodeling, which are often disrupted in the diabetic microenvironment. Hydrogels have emerged as promising solutions for DFU management by incorporating antimicrobial agents, growth factors, and immunomodulatory components; hydrogels can accelerate healing by regulating inflammation, promoting angiogenesis, and enhancing tissue regeneration, offering a targeted and efficient therapeutic approach for DFUs. Table 3 provides advanced hydrogels engineered for target-specific phases of DFU healing, with their in vivo outcomes and therapeutic efficiency. This review focuses on the categories of hydrogels employed in treating a DFU across the various stages of wound healing.

### 5.1. Hydrogels for Inducing Hemostasis

As discussed in the stages of wound healing, it is during the hemostasis phase that the body prepares for wound closure by employing mediators of wound closure like PDGF and TGF-β. Thus, these growth factors play a vital role in orchestrating this phase by regulating vasoconstriction, platelet activation, and clot formation. Since the deregulation of these growth factors is a hallmark of chronic wounds, addressing this deregulation by growth-factor-induced hydrogels presents a promising avenue for treatment. Targeting and sustained release can be achieved by encapsulating growth factors within a biomimetic hydrogel matrix, potentially preventing the transition to the inflammatory phase. Because platelets mediate between the vascular system, hemostasis, and the immune system, their roles in wound healing differ at each process stage. Platelets are present throughout. The activated platelet granules include many growth factors (GFs), chemokines, and cytokines involved in angiogenesis and the recruitment, proliferation, and differentiation of injured cells. These granules aid in coagulation through the action of proteins. Hydrogels are a potential delivery system for the regulated release of bioactive molecules like proteins, GFs, and enzymes for tissue regeneration. Hydrogels facilitate cell adhesion and resemble the natural extracellular matrix. Their hydrophilic quality produces a favorable microenvironment for cell proliferation [14].

Platelet-rich plasma (PRP), rich in growth factors like TGF-β and VEGF, offers a bioactive matrix for wound healing by promoting tissue regeneration and reducing inflammation [115]. PRP hydrogels, incorporating essential growth factors, enhance wound repair by stimulating tissue regeneration and reducing inflammation. Recent studies developed composite hydrogels using silk fibroin, glycol chitosan, and PRP, demonstrating controlled degradation and sustained bioactive factor delivery [115]. Composite hydrogels combining silk fibrin, chitosan, or hyaluronic acid with PRP have shown enhanced antibacterial activity, controlled degradation, and reduced inflammatory markers, especially in diabetic wounds. While in vitro studies report improved cell proliferation and adhesion, in vivo outcomes vary based on PRP source. However, limitations such as rapid growth factor release and the need for frequent application reduce therapeutic efficiency and patient comfort [116].

A recent study developed composite hydrogels incorporating PRP with additional stabilizing agents such as DPLG (Deferoxamine + PRP + Laponite) gel to address these limitations. The hydrogel enhances hemostasis by promoting clot stability. Compared to PRP gel alone, DPLG significantly accelerated wound closure in the diabetic rat model, with only 18.9% of the wound area remaining by day 9, in contrast to 63.4% in the control group [31,117]. Blood plasma emerges as a highly promising material for developing bioactive structures owing to its structure and autologous nature, derived from the same individual [118]. In the intricate process of blood clotting, platelets play a pivotal role. Upon the release of their granules, platelets exhibit a remarkable ability to induce tissue repair, regenerate blood vessels, and facilitate cellular differentiation [119].

PRP represents a reservoir of platelets exhibiting a high concentration. It serves as a natural sealant and carrier for indispensable platelet-derived growth factors, encompassing platelet-derived growth factor (PDGF), TGF-β, PF4, interleukin-1(IL-1), PDAF, VEGF, EGF, ECGF, and IGF. These factors play a crucial role in the promotion of wound healing by fostering the active involvement of neighboring cells in tissue repair while concurrently regulating cytokine release and macrophage activity, leading to a reduction in inflammation and an acceleration in tissue regeneration, particularly in the context of conditions such as DFUs [120,121]. Recent investigations have demonstrated the successful incorporation of PRP into hydrogel dressings for the treatment of DFUs, exemplified by the work of Qian et al., in which a composite hydrogel employing glycol chitosan, silk fibroin, and PRP was subsequently evaluated using a diabetes type-2 rat model. In their approach, polyethylene glycol (PEG) was chemically modified with 4-carboxybenzaldehyde (CB), thereby employing it to crosslink glycol chitosan by a reversible Schiff base reaction mechanism [122]. Furthermore, silk fibroin was integrated into the formulation to enhance the mechanical strength of the composite hydrogel. The resulting composite hydrogel exhibited a controlled degradation of PRP and a sustained release of bioactive agents, surpassing the performance of the pure PRP hydrogel. This enhanced performance could be attributed to the inherent resistance of chitosan and silk fibroin to enzymatic degradation. In a study conducted by Shah et al. on bioactive polymers like Chondroitin Sulfate (CS) and Sodium Alginate (SA) were assessed to test their cytocompatibility, physicochemical properties, and antibacterial efficiency. SA drives cellular processes like adhesion and proliferation, demonstrates hemostatic properties, and lowers the bacterial load at the site of infection. In addition, wound exudate is retained because of its hydrophilic properties, optimizing the wound microenvironment and efficiently enabling the wound healing cascade. SA is known for its hemostatic and antibacterial properties. It also enhances collagen synthesis, contributing to the tensile strength required for skin growth. In this study, streptozotocin-induced diabetic rats were used as models to study the wound healing process by loading curcumin-infused hydrogels developed in situ for targeted and controlled delivery onto the diabetic wound environment. It was shown that curcumin is involved in the stimulation of TGF-β1, which is an essential component during platelet activation. This hydrogel was reported to enhance re-epithelialization, increase granulation tissue, angiogenesis, collagen deposition, and improve extracellular matrix composition necessary for wound healing [123].

### 5.2. Hydrogels Targeting Inflammation

The inflammation process follows Hemostasis as it serves several essential functions in the healing process by clearing cell debris, dead cells, and pathogens from the wounded area, preventing infection, and promoting a clean environment for healing. Neutrophils, mast cells, and macrophages initiate the inflammation reaction. These cells are responsible for the production of the inflammatory cytokine interleukin 1 (IL-1), tumor necrosis factor-alpha (TNF-α), interleukin 6 (IL-6), PDGF, and epidermal growth factor (EGF) [14]. The surrounding wound tissue releases these proinflammatory cytokines and growth factors. At the same time, the neutrophils clear the cellular debris from the wound area and kill the microorganisms by generating reactive oxygen species (ROS). Early on, macrophages release cytokines to boost the immune response by bringing in and activating more leukocytes. Additionally, macrophages trigger cell death and eliminate dying cells and neutrophils [124]. Similarly, Wei et al. developed a hydrogel with enhanced antibacterial activity and inflammation-regulating capabilities specifically designed for the diabetic wound environment [125]. The formulation utilized hyaluronic acid (HA) and dextran as the base polymers, which were suitably chemically modified to allow for the attachment of an antimicrobial peptide (AMP) named cecropin to HA. In contrast, dextran was functionalized with aldehyde groups to facilitate crosslinking and hydrogel formation. The hydrogel demonstrated promising antibacterial properties, effectively curbing the proliferation of infectious agents in diabetic wounds and exhibiting a considerable reduction in inflammatory factors [126]. Xiaoliang et al. have addressed the issue of ROS and inflammatory cytokines that hinder the macrophage transition from M1 to M2 cell migration, causing prolonged inflammation. They developed an immunomodulatory hydrogel (PHG2) composed of platinum-deposited epigallocatechin-3-gallate (Pt@EGCG) nanoparticles, polymers such as thiol-modified gelatin (Gel-SH), and phenylboronic acid-modified hyaluronic acid methacryloyl (HAMA-PBA) to reduce the inflammatory phase by targeting the ROS reduction. Epigallocatechin-3-gallate nanoparticles are proven for ROS scavenging activity and also act as a base material to attach platinum nanoparticles. These platinum nanoparticles show catalase-like functionality that helps convert hydrogen peroxide into water and O_2_. The hydrogel showed ROS scavenging as well as O_2_ generation. Increased ROS clearance and elevated O2 levels were associated with the M1 to M2 polarization, as evidenced in RAW 264.7 macrophages, RS1 cells, and diabetic rats when treated with the developed immunomodulatory hydrogel [127].

MXenes are an emerging category of 2D nanomaterials that show distinct physicochemical and structural characteristics. Applying a polydopamine (PDA) coating onto MXene has been found to augment its antibacterial and antioxidant properties and promote the interconnection of nanosheets into a hydrogel network. Consequently, the resulting hydrogel can modulate the polarization of macrophages from the M1 to the M2 phenotype, thereby leading to a notable anti-inflammatory effect. Recent investigations have revealed that MXenes exhibit robust antioxidant, anti-inflammatory, and antibacterial attributes. These advancements have significantly propelled the progress of MXenes in tissue engineering and skin regeneration. Incorporating MXenes into wound dressings has revolutionized novel research in wound healing through a series of innovative studies. The presence of hypoxia, reduced oxygen supply, elevated levels of reactive oxygen species (ROS), impaired neovascularization, prolonged inflammation, and bacterial infection creates obstacles that hinder the healing of wounds in diabetic patients. Notably, the control of oxygen release and the elimination of ROS are crucial during the wound healing process. The stable thermal response exhibited by MXene ensures the consistent release of oxygen. Additionally, the utilization of MXene nanosheets as synthetic nonenzymatic antioxidants for the eradication of undesired reactive oxygen species (H_2_O_2_, O_2_^•−^, and ^•^OH) and reactive nitrogen species (•NO, •NO_2_) has been studied by researchers. This scavenging function can reduce oxidative stress, prevent bacterial infection, and support intracellular redox homeostasis. An MXene-anchored hydrogel possesses hemostasis, self-healing, tissue adhesion, and injectability properties. When combined with mild photothermal stimulation, this hydrogel significantly enhances the proliferation and migration of human umbilical vein endothelial cells, thereby greatly facilitating the healing of infected diabetic wounds [128].

Novel Au–Pt nanozyme-based self-healing hydrogel dressing (OHCN) is developed for diabetic wound healing. This dressing addresses the complex microenvironment of diabetic wounds, combining Au-Pt alloy nanoparticles with an OHC hydrogel. The OHCN hydrogel exhibits antibacterial properties, self-healing capability, and efficient regulation of pathological conditions, including glucose reduction and ROS elimination. Through synergistic effects, it accelerates diabetic wound healing by improving the wound microenvironment. This multifunctional hydrogel presents a promising strategy for enhanced diabetic chronic wound management, offering self-repair, antibacterial action, glucose level regulation, ROS elimination, and O_2_ release [129]. Zinc oxide nanoparticles (ZnO) are explored as potent antibacterial agents due to their ability to generate reactive oxygen species (ROS). To address the challenges of ROS production in the body, catechol-ZnO complexes were incorporated into a hyaluronic acid (HA) hydrogel. This innovative hydrogel demonstrated enhanced ROS production, offering effective antibacterial properties. The hydrogel exhibited unique physical characteristics, including increased swelling ratio, enzymatic degradation resistance, and tissue adhesive strength [130].

Particle hydrogels (PHs) synthesized from precursors, such as polymethylmethacrylate (PMMA) and PVA, offer a versatile platform for designing wound dressings with tailored properties. Acid-responsive components, including poly-methacrylic acid (PMAA) and polyacrylic acid (PAA), further enhance the adaptability of PHs in response to the specific needs of diabetic wounds. The synthesis of PHs involves a crosslinking reaction with ultraviolet (UV) light, forming a reticular structure. The choice of precursors and the synthesis technique allows for the regulation and control of the size of PHs. Additionally, the diameter of the syringe during synthesis plays a crucial role in determining the size of these hydrogels, providing a means to tailor their characteristics for specific applications such as controlled drug release [131]. The hydrogel undergoes polymer-polymer bonding and shrinking in acidic conditions (pH 3.0). In contrast, under weakly alkaline conditions (pH 8.0), the hydrogel swells significantly, mimicking the conditions of diabetic wounds and promoting drug release. This pH-dependent behavior allows precise control over drug release rates, with implications for inhibiting bacterial infection in the early stages of diabetic wounds [132]. Silver nanowires, known for their antibacterial properties, are incorporated into PHs to enhance their therapeutic capabilities. This study explores the antibacterial abilities of PHs containing silver nanowires with varying concentrations (175 ppm and 350 ppm). Intriguingly, wound healing in the 350-ppm silver nanowire group during inflammation outperforms the 175-ppm group. This study suggests that in the early stages of wound inflammation, higher concentrations of silver nanowires may contribute to more effective antibacterial outcomes [131].

The chronicity associated with DFUs can be attributed to various factors, with oxidative stress standing as a predominant damaging element affecting diabetic ulcer healing. Consequently, extensive research is currently focused on developing hydrogels with scavenging activity that help protect the wound bed from the detrimental effects of reactive oxygen species (ROS) on mitochondrial function. Considering this, Xu et al. conducted a recent study involving the development of a poly(d, L-lactide)-PEG-poly(d, L-lactide) (PLGA-PEG-PLGA) hydrogel that could encapsulate and release Prussian blue nanoparticles (PBNPs). PBNPs are synthetic materials that imitate the ROS-scavenging activity of biological antioxidant enzymes, namely superoxide dismutase, peroxidase, and catalase. The thermosensitivity of the developed hydrogel facilitated the in situ gelation at physiological temperatures [133]. Moreover, in vivo studies conducted on diabetic murine wound models provided evidence of the scavenging activities of PBNPs, as validated by dihydroethidium staining. This led to an anti-inflammatory response, effectively reducing chronic inflammation and improving wound healing and angiogenic remodeling compared to the control group without PBNPs.

### 5.3. Hydrogels Promoting the Proliferation of Cells in the Wound Site

Hydrogel-containing PRP demonstrated increased vascular endothelial growth factor (VEGF) expression, confirming its ability to promote angiogenesis. The biodegradability of the hydrogel could be judiciously modulated by altering the quantity of sodium periodate (NaIO_4_) employed for the oxidation of alginate [115]. Exosomes (EXOs), nano-sized lipid bilayer vesicles crucial for physiological regulation, hold promise as disease biomarkers and therapeutic agents [134]. Exosomes, harnessed for diabetic wound healing, are encapsulated within hydrogels to exploit their vascular regenerative, anti-inflammatory, and nerve regeneration effects. Researchers employ innovative compositions, such as pH-responsive carboxymethylcellulose hydrogels loaded with plasma exosomes, increasing angiogenesis and re-epithelialization [135]. Hydrogels combining GelMA/PEGDA, tazarotene, and HUVEC exosomes accelerate vascular endothelial cell proliferation [136]. Moreover, hydrogels incorporating M2-derived exosomes and FGF-2 synergistically induce angiogenesis and tissue regeneration [137]. Loading hydrogels with stem cells like endothelial progenitor cells enhances HIF-α levels, initiating angiogenesis and aiding in wound healing. These advancements demonstrate the potential of exosome-loaded hydrogels for multifaceted diabetic wound therapy.

Hydrogels incorporating metal and metal-derived nanoparticles and nanoclay are promising hydrogels to promote angiogenesis in the proliferating phase. Carboxymethyl chitosan (CMCS) was covalently linked to protocatechualdehyde (PCA) to create self-healing hydrogels through amide bond formation. Ultra-small copper nanoparticles (CuNPs) were incorporated into the CMCS-PCA hydrogel, and their structure was confirmed via XRD, TEM, and XPS analyses. The resulting CuNPs@CMCS-PCA hydrogel exhibited self-healing capability. In vitro and in vivo assessments highlighted the therapeutic potential of CuNPs@CMCS-PCA in diabetic wound healing. The primary role of copper in promoting VEGF expression, enhancing endothelial cell growth, and facilitating angiogenesis was elucidated, emphasizing its applicability in wound healing [138]. These hydrogel nanocomposites are created by introducing synthetic 2-D nanoclay into a chitosan or gelatin polymer matrix. Laponite, chemically known as NaO_7_(Mg_5_.5LiO_3_) Si_8_O_20_(OH)_4_, is a commercially recognized nanoclay composed of two-dimensional disc-shaped, stacked silicate sheets measuring 25 nanometers in diameter and a mere 1 nanometer in thickness. These are held together by weak van der Waals forces, enabling them to glide easily past one another [139]. Notably, it features negative charges distributed on its faces (OH^−^) and positive charges on its edges (Na^+^). This feature enables efficient dispersion at low concentrations in an aqueous environment. The gel-forming capability of Laponite follows a multi-step mechanism. Initially, when particles interact with hydroxide ions in water, phosphate ions dissolve. Subsequently, nanoclay particles engage in interparticle interactions, while sodium ions diffuse toward the surfaces within the galleries [140]. This intricate process results in the formation of an expanded thixotropic gel-like structure. Morairu et al. conducted a study where the incorporation of laponite into polyvinyl alcohol hydrogels was found to enhance their characteristics, including structural regeneration (approximately 90%), water absorption (up to 900%), and antibacterial activity. Moreover, the release of rifampicin from these hydrogels was contingent on the concentration of the clay [141]. Howell et al. investigated the use of laponite in collagen type I hydrogels loaded with proangiogenic proteins, including vascular endothelial growth factor, fibroblast growth factor, and platelet-derived growth factor. This formulation significantly stimulated endothelial cell sprouting, leading to improved angiogenesis, accelerated tissue repair, and a reduced risk of chronic, non-healing ulcers [142]. This approach holds promise for addressing the intricate challenges associated with healing DFUs in individuals with diabetes.

Additionally, incorporating clay particles, particularly laponite, into gelatin resulted in the development of stable clot-gel systems characterized by enhanced physiological stability, rapid structure recovery, and the ability to promote coagulation. Consequently, a substantial 77% reduction in vitro blood clotting time was observed [143]. Furthermore, the integration of Na-MMT (montmorillonite) nanoclay particles into polyvinyl alcohol hydrogels was shown to serve as an effective barrier against microbial infiltration. Thus, it contributed to heightened defense against subsequent wound infections and expedited wound healing [144]. These hydrogels protected against skin infections and fostered an environment conducive to efficient wound healing. While these properties make nanoclay an appealing choice, hydrogels printed using it often exhibit high viscosity and form stiff networks. These challenges must be addressed to fully harness their advantages in wound healing. Gelatin, derived from collagen, is a naturally occurring polymer extensively investigated for its potential application in wound dressings. The unique properties of gelatin make it an excellent carrier for bioactive molecules like growth factors and cytokines that are produced by mesenchymal stem cells. Yoon et al. showed that the chemokine-loaded sprayable gelatin hydrogel dressing material can recruit the cells to the wound area [145]. These bioactive agents promote tissue repair and healing by modulating the wound microenvironment and enhancing various cellular processes, including migration, adhesion, and proliferation. Chen et al. observed in their study that DFO-loaded photo-crosslinked gelatin hydrogels accelerate angiogenesis. This is particularly important in conditions like DFUs, where impaired wound healing is a significant concern [146]. It ensures the delivery of oxygen and nutrients to the site, which is essential for proper wound healing.

### 5.4. Hydrogels Promoting the Remodeling of Wound Tissue

Silicone, also known as polysiloxane, is a versatile biomaterial synthesized by hydrolyzing chlorosilanes with water, along with silanols like dihydroxysilane and chlorohydroxysilane, followed by dehydration and dehydrochlorination. This process results in polymers that exhibit remarkable adaptability, transforming them into various materials suitable for different applications, including flexible materials, gels, adhesives, and more. One notable property of silicone is its very low glass-transition temperature, approximately −125 °C, enabling flexibility in extremely cold conditions, such as those encountered in cold storage, due to its ability to maintain physical properties between −40 °C and 185 °C. A study investigated the efficacy of silicone gel in promoting faster epithelialization and secondary intention (SI) wound healing following the removal of skin tumors. This study focused on elderly individuals with tumors on their scalps or extremities. Each incision was treated with silicone gel, an internal purse-string suture, and a standard paraffin gauze dressing, allowing the wounds to heal naturally through SI. The mechanism of action of silicone gel in wound healing involves creating an optimal environment for the healing process. Silicone gel maintains a humid climate while regulating moisture vapor transmission, which is crucial to prevent dehydration and overhydration that facilitates epithelialization, an essential step in wound closure, while ensuring a controlled rate of moisture vapor transmission to avoid complications associated with excessive moisture [147]. Silicone’s flexibility, adaptability, and unique properties make it a promising candidate for diabetic wound care. Further research and development may enhance the formulation of silicone-based dressings, addressing limitations and expanding their role in promoting effective wound healing, particularly in the challenging context of DFUs.

Carbon nanomaterials have attracted significant attention due to their remarkable properties. Graphene oxide (GO) and its reduced form, carbon quantum dots (CQDs), carbon nanofibers (CNFs), carbon nanotubes (CNTs), and nanodiamonds (NDs) are a few of the carbon nanomaterials that are being studied with respect to wound dressing applications [148]. Carbon nanotubes are nanoparticles with various properties, such as physicochemical and conductive properties of carbon nanomaterials, allowing them to be extensively studied. As we all know, the wounded area is moist and more susceptible to infections. CNTs can induce ROS, such as O_2_- and OH, which can kill the bacteria, thus exhibiting antibacterial properties. The treatment of various types of wound healing can be achieved with a variety of carbon-based nanocomposites, which have several beneficial properties like enhanced oxygen permeability, reduced wound healing time, hemocompatibility, antibacterial properties, and cell adhesion have been reported. CNT hydrogels are associated with most of the features of hydrogel polymeric networks. The production of CNT hydrogel hybrid is generally made possible by five polymerization techniques: (i) covalent cross-linking (in-situ polymerization), (ii) ex-situ polymerization, (iii) physical cross-linking, (iv) polymer grafting, and (v) smart devices [149]. Gelatin-based hydrogel dressings have been found to facilitate the formation of granulation tissue and epithelial tissue, both of which are critical for the successful treatment of diabetic wounds. The ability of gelatin-based hydrogels to support the generation of these essential tissues makes them highly valuable in wound healing. In addition to traditional gelatin-based hydrogels, researchers have also developed an innovative microneedle patch system using a composite matrix of GelMA and PEGDA [136]. This system offers a unique advantage regarding the transdermal delivery of biological agents such as tazarotene. Unlike traditional drug delivery methods involving injections, the microneedle patch system allows for pain-free administration of therapeutic agents. This innovation holds great promise for improving patient compliance and overall treatment outcomes.

Furthermore, researchers have explored the use of gelatin-hyaluronic acid composite hydrogels in diabetic wound healing. These hydrogels are created using EDC chemistry and impregnated with recombinant thrombomodulin (rhTM). Incorporating rhTM in the hydrogel has been found to stimulate collagen generation, re-epithelialization, granulation tissue formation, and angiogenesis in diabetic wounds. These effects can be attributed to the hydrogels’ self-recovery property and microporous structure, which facilitate cell adhesion and migration, further enhancing the wound-healing process. In conclusion, gelatin-based hydrogels have emerged as a promising option for wound dressings in wound healing. Their unique properties, such as the ability to serve as carriers for bioactive agents and the ability to promote the formation of essential tissues, make them highly valuable in treating diabetic wounds. The development of innovative systems, such as the microneedle patch and composite hydrogels, further expands the potential applications of gelatin in wound healing. The Future research in this area holds great promise for improving patient outcomes and advancing the field of wound healing. 

PEG-based hydrogels have become a favored choice for biological systems for their notable biocompatibility and ability to resist protein adhesion [150]. The incorporation of functional groups into these hydrogels opens the possibility of creating derivatives such as PEGDM and PEGDA, which can undergo chemical crosslinking to generate robust matrices capable of incorporating biomolecules that promote tissue regeneration [151]. Among the vast array of synthetic biomaterials available, PEG and its derivatives have been extensively utilized to fabricate biocompatible hydrogels for cell culture scaffolds and tissue engineering applications. One of the main reasons PEG-based hydrogels have been widely employed in developing biological systems is their remarkable biocompatibility and resistance to protein adhesion, which are achieved by incorporating functional groups. Xu et al. investigated wound healing by creating an in situ polymerizable hydrogel to encapsulate adipose-derived stem cells, thereby enhancing skin regeneration in a murine diabetic wound model [152]. Hyperbranched multi-acrylated PEG macromers were prepared using a reversible addition−fragmentation chain-transfer (RAFT) polymerization mechanism. These macromers were subsequently thiolated with hyaluronic acid to form a hydrogel through a thiol-ene click reaction. The results obtained from the in vivo testing demonstrated the therapeutic efficiency of this proposed system, as indicated by the increased healing rates and formation of granular tissue.

Stem-cell-exosome-loaded hydrogels have shown the ability to inhibit the differentiation of myofibroblasts, a significant contributor to excessive scar formation during the remodeling phase. In a study using a full-thickness dorsal wound model in mice, Adipose-derived stem cells (ADSCs)-derived exosomes were administered intravenously, resulting in a significant reduction in scar formation through the modulation of fibroblasts. These exosomes enhanced the ratio of transforming growth factor beta 1 (TGFβ1) to TGFβ3, inhibiting collagen types I and III expression in fibroblasts. This inhibited their myofibroblast differentiation and decreased granulation tissue formation. Moreover, ADSC-exosomes in vitro activated the MAPK/ERK pathway in skin dermal fibroblasts, increasing the matrix metalloproteinase 3 (MMP3) ratio to tissue inhibitor of metalloproteinases 1 (TIMP1). This increase promotes scarless skin repair through ECM remodeling [153].

Adipose-derived stem cell (ADSC)-loaded hydrogels, specifically hydrogels derived from acellular porcine adipose tissue (HAPA), have been shown to enhance wound healing by promoting closure, collagen turnover, and formation of an organized ECM. This prevents excessive fibrosis and improves the structural stability of regenerated skin. Other factors and cytokines released by adipocytes, such as vascular endothelial growth factor (VEGF) and platelet-derived growth factor (PDGF), promote angiogenesis, ECM remodeling, and fibroblast-mediated tissue repair. Interestingly, ADSC-loaded HAPA-treated wounds showed increased regeneration of skin appendages such as hair follicles and sebaceous glands, illustrating the promise of the approach for long-term skin remodeling and regeneration. Furthermore, ECM remodeling via neovascularization is essential for re-establishing normal vascular integration in newly created tissue. ADSC-loaded hydrogels greatly improved neovascularization, as indicated by the enhanced expression of CD31 and release of VEGF in wound models [154].

**Table 3:** Advanced Hydrogels Designed for Targeting Specific Phases of DFU Healing: An Overview of Composition, Hydrogel Type, and Therapeutic Benefits.

Therapeutic Phase	Material Composition	Hydrogel Type	Preclinical/ Clinical Data	Therapeutic Efficacy	Reference
Inflammatory, Proliferative	*Bletilla striata* polysaccharide (BSP) + Berberine + Borax	Self-healing injectable hydrogel	In vivo (DFU mouse) ~95% healing in 14 days	- Hemolysis rates < 5%, - Cell mobility rate of 48.17 ± 1.68% - Anti-inflammatory (BSP/BER10 hydrogel reduced MCP-1 by 85.5%, IL-6 by 40.5%, and TNF-α by 83.7%)	[155]
Inflammatory	Chitosan + β-CD + Trans-Cinnamaldehyde	Injectable hydrogel	In vitro only (antibacterial, antibiofilm)	- Injectable, cytocompatible - 99.99% antibacterial - 58–60% antibiofilm	[156]
Inflammatory, Early Proliferation	Carboxymethylcellulose (CMC) + Green-synthesized AgNPs	pH-responsive hydrogel	In vitro: ~75% wound closure; ex vivo enzyme inhibition	- Biocompatible - Sustained AgNP release - 84% MPO and 73% collagenase inhibition - Angiogenesis and collagen promotion	[157]
Hemostasis, Inflammation	Alginate + Hyaluronic Acid + Zn^2+^ + Polydopamine (Alg–HA–Zn–PDA)	Photosensitive hydrogel	In vivo (rat tail-amputation and infected full-thickness wound model) and in vitro BCI and scratch assay; ~69% cell migration, accelerated wound closure	- Hemostatic time (<30 s vs ~5 min untreated) - Blood loss (0.3 g vs 1.5 g untreated) - Antioxidant (DPPH scavenging ~80%) - Enhanced fibroblast migration (69.15%)	[158]
Inflammation, Proliferation	Chitosan + Ag^+^ + EGF-loaded nanoparticles	pH-responsive	In vivo (diabetic rat): 97% healing	- Multifunctional: antibacterial, healing, anti-inflammatory - Oxygen delivery (PFC) - Sustained release in wound environment	[159]
Inflammation, Proliferation, Remodeling	Chitosan + PVA + PHMB + EGF-loaded NPs + Perfluorocarbon emulsions	pH-responsive	In vivo (diabetic rat): 95% healing in 15 days	- Multifunctional: antibacterial, healing, anti-inflammatory - Oxygen delivery (PFC) - Sustained release	[160]

## 6. Challenges and Future Perspectives

The review highlights the complex challenges in treating DFUs. Key issues include impaired wound healing processes due to hyperglycemia, chronic inflammation, hypoxia, altered pH, and increased susceptibility to infections. The review emphasizes the need for multifunctional hydrogels that can address multiple aspects of diabetic wound healing simultaneously. Despite progress, various challenges continue to impede their use in clinical settings. Designing scaffolds for treating DFUs presents intricate issues that significantly affect clinical results. The main difficulty is choosing the right material that suits the DFU microenvironment. Existing patches mainly target the early stages of DFU, concentrating on addressing problems like blood clotting, exudate absorption during infections, and the inflammatory phase. There is an urgent demand for hydrogel patches that can adapt to the changing wound environment throughout the healing process, including tissue remodeling and regeneration. Hydrogels fabricated with natural polymers, such as collagen, gelatin, and chitosan, have good biocompatibility and bioactivity, but also have poor mechanical strength and degrade quickly. It is difficult to control the pace of degradation and the release of growth factors and drugs. Because DFUs heal slowly, scaffolds need to break down in time with tissue growth. Failure can result from too-slow degradation, leading to fibrosis or inflammation. Synthetic and composite polymers have drawbacks such as cytotoxicity, poor biocompatibility, poor bioactivity, poor environmental degradability, and cost, which restrict their use in biomedical applications. Therapeutic release that is sustained and localized is essential, but it depends on surface chemistry, porosity, scaffold architecture, tunable mechanical properties, and swelling and degradation kinetics—all of which are challenging to regulate precisely. Designing stimuli-responsive multifunctional hydrogels using nanomaterials offers a significant advantage in modulating the DFU microenvironment. The other challenge includes the availability of biomimicking in vitro models to study the DFU microenvironment and mechanisms underlying wound healing. Therefore, using 3D culture systems incorporating microfluidic technology, the DFU microenvironment can be recapitulated in vitro.

Two of the biggest technological obstacles to bringing scaffolds for treating DFU to clinical application are scalability and cost-effectiveness. Large-scale production of promising scaffold designs for DFU treatment faces challenges despite preclinical success. Current fabrication techniques offer precise control but are labor-intensive, time-consuming, and costly, limiting mass production potential. Balancing high-quality designs with scalability remains a significant hurdle. Researchers are exploring innovative approaches to streamline production and reduce costs without compromising efficacy. This includes investigating alternative materials, developing automated processes, and optimizing fabrication techniques. Collaborative efforts between academia and industry aim to bridge the gap between laboratory success and commercial viability. One of the goals of biomaterial research is to introduce sensor-based, electroconductive hydrogels to sense wound healing efficiency and release the drug to the targeted wound without toxicity and poor mechanical properties. In the future, the construction of high-quality, biocompatible, non-toxic wound-healing hydrogel patches can have great market value in the current and coming decades. However, significant advancements in manufacturing technologies and cost-reduction strategies are still needed to overcome current limitations and bring effective scaffolds to widespread clinical use. Continued research and innovation in this area can significantly improve outcomes for patients with this challenging condition.

## 7. Conclusions

Diabetes is escalating at an alarming rate and is now considered a global epidemic, with millions of new cases diagnosed each year. It is concerning that complications associated with diabetes can put diabetic individuals at risk when it comes to wound management. Among its severe complications, DFUs present a significant challenge due to impaired and delayed wound healing. These chronic wounds are difficult to manage clinically because of their complex microenvironment. In contrast, conventional treatments offer limited effectiveness. Alongside clinical treatment, cutting-edge therapeutic strategies are tailored to the pathophysiological phases of diabetic wound healing. Hydrogels have gained attention as a versatile and effective wound management solution. Their advantageous features include versatile surface chemistry, polymer types, tunable mechanical properties, and slower degradation, allowing the design of smart dressings that adapt to wound-specific conditions. In particular, stimuli-responsive hydrogels enable controlled drug release and modulate wound healing. Moreover, hydrogels that are designed to function in a phase-dependent manner, incorporating therapeutic agents that are selectively released based on the phases of wounds, hemostasis, inflammation, proliferation, and remodeling stages, are a promising strategy for developing next-generation wound healing patches specifically suited for managing diabetic wounds.

## Figures and Tables

**Table 1:** Commercially available hydrogels for the treatment of diabetic and chronic wounds, with their target wound healing phase.

Product	Company	Targeting Phases	Polymers Used
SERIDERM^®^	Serigen Mediproducts Private Limited, Pune, India	Inflammatory phase (Absorption of exudate)	Silk protein
AQUACEL^®^ Hydrofiber^®^ Dressing	ConvaTec Group Public Limited Company, London, UK	Inflammatory phase (Absorption of exudate)	Sodium carboxymethylcellulose
RMD-G1 (a hydrogel containing erythropoietin)	Remedor Biomed Limited, Nof HaGalil, Israel	Proliferative phase (Rapid development of granulation tissue)	Carbopol-based hydrogel
NanoDOX™ Hydrogel	Nanopharmaceutics, Inc., Alachua, FL, USA	Proliferative phase, Proangiogenic phase (Growth of new blood vessels)	PEG, Polyvinyl alcohol (PVA), Natural polymers (Chitosan, gelatin, and alginate).
Cadexomer iodine gel	Smith and Nephew, Watford, UK	Infection stage (Release of iodine)	Starch-derived polymer beads, Iodine iodophore
Regranex^®^	Smith and Nephew, Watford, UK	Apoptosis, Proliferative stage. Angiogenesis	Carboxymethylcellulose Sodium, Methylparaben.
ConvaTec DuoDERM Hydroactive Gel	ConvaTec Group Public Limited Company, London, UK	Proliferative stage (Granulation Tissue Formation)	Carboxymethyl cellulose, Pectin, Gelatin, Methylparaben, and Propylparaben
IZN-6D4 Gel	Izun Pharmaceuticals, New York, NY, USA	Proliferative Stage	Carboxymethyl cellulose and PVA.
XCell^®^	Xylos Corporation, Langhorne, PA, USA	Inflammatory and proliferative stage.	Bacterial nanocellulose
Dermafill™	Cellulose Solutions, LLC, Daphne, AL, USA	Inflammatory stage	Bacterial Nanocellulose

**Table 2:** Advantages and disadvantages of natural, synthetic, and composite hydrogels in DFU treatment.

Type	Advantages	Disadvantages	References
**Natural Hydrogels** (e.g., Alginate, chitosan, collagen, hyaluronic acid, gelatin, fibrin)	- Imitates native extracellular matrix, Supporting cell adhesion, proliferation, and migration. - Intrinsic bioactivity promotes angiogenesis and tissue repair - Is biocompatible and biodegradable - Has the ability to recruit and modulate immune cells for wound healing - Generally low to no cytotoxicity	- Poor mechanical strength, prone to degradation under stress. - Variability from batch to batch due to biological origin - Shorter shelf life and limited storage stability - Immune response and contamination risk	[67,80,82,83]
**Synthetic Hydrogels** (e.g., PEG, PVA, polyacrylamide, PLA, PLGA, Pluronic F127)	- Tunable chemical and mechanical characteristics allowing customization for different wound environments. - Stable and highly reproducible with controlled composition. - Longer shelf life and better storage stability. - Can be functionalized with bioactive molecules for drug delivery - Show high mechanical strength in hydrogels - Reduced risk of contamination - Has slower degradation	- Lack intrinsic bioactivity, often requiring functionalization to promote cell adhesion - Possible cytotoxicity from unreacted monomers or degradation of polymer - Have limited integration with the host tissue without modification	[70,74,84]
**Composite Hydrogels** (Natural + Synthetic e.g., Chitosan-PEG, alginate-PVA, collagen-PLC, Nanoclay, Nanofibers)	- Combines the stability and mechanical strength of a synthetic polymer with the bioactivity of a natural hydrogel. - Improved mechanical strength and elasticity - Improved ECM mimicry, enhancing cell proliferation and migration. - Enables controlled release of therapeutic agents - Suitable for stimuli-responsive hydrogels	- Complex fabrication - High production cost and time-consuming optimization - May require expensive or time-consuming optimization - Stability issues during storage - Require extensive validation for regulatory approval (safety, quality, and efficiency)	[85,86]

## Data Availability

No primary research results, software, or code have been included, and no new data were generated or analyzed as part of this review.

## Supplementary Materials

The following supporting information can be downloaded at: https://www.mdpi.com/article/10.3390/polym17172303/s1, Figure S1: Stages of Diabetic Foot Ulcer Development to Gangrene.
