# Supplementary material for: Progressive Hydrogel Applications in Diabetic Foot Ulcer Management: Phase-Dependent Healing Strategies
Source: Polymers (Basel). 2025 Aug 26;17(17):2303. doi: 10.3390/polym17172303 (PMC12431415; doi:10.3390/polym17172303)
Supplement: Supplementary file 1 [file polymers-17-02303-s001.zip › polymers-3749431-supplementary.pdf]

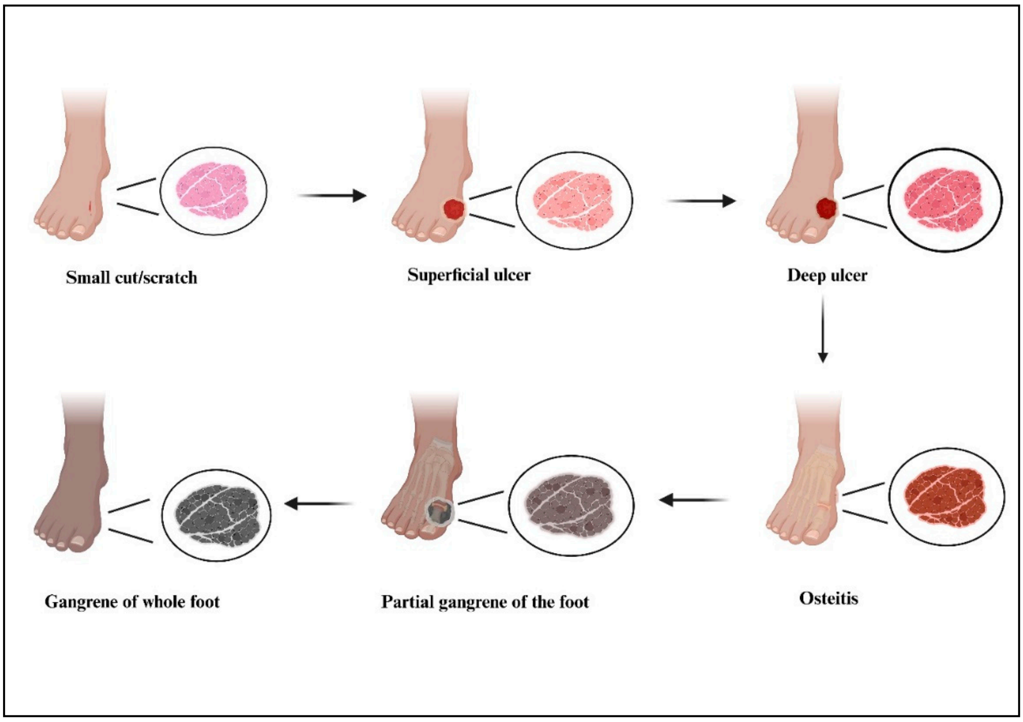

Supplementary figure S1. Stages of Diabetic Foot Ulcer Development to Gangrene (Image source: Created with BioRender.com).
